# Environmental Burden of Diesel Engines: A Life‐Cycle Assessment and the Imperative for Cleaner Alternatives

**DOI:** 10.1002/gch2.70130

**Published:** 2026-07-16

**Authors:** Sanjeev Kumar, Shams Forruque Ahmed, Amit Pal, Minh Thien Nguyen, Thanh Hai Nguyen, Minh Thai Duong, Duc Chuan Nguyen, Hoang Dat Do, Dao Nam Cao

**Affiliations:** ^1^ Department of Mechanical Production & Industrial Engineering Delhi Technological University Delhi India; ^2^ School of Mathematical Sciences Sunway University Petaling Jaya Selangor Darul Ehsan Malaysia; ^3^ Miyan Research Institute International University of Business Agriculture and Technology Dhaka Bangladesh; ^4^ Institute of Research and Development Duy Tan University Da Nang Vietnam; ^5^ School of Engineering & Technology Duy Tan University Da Nang Vietnam; ^6^ Energy‐Fuel Technology and Applied Material Research Group Dong Nai Technology University Dong Nai Vietnam; ^7^ Institute of Research and Application of Technology and Science Dong Nai Technology University Dong Nai Vietnam; ^8^ Institute of Mechanical Engineering University of Transport Ho Chi Minh city Ho Chi Minh City Vietnam; ^9^ Institute of Maritime University of Transport Ho Chi Minh city Ho Chi Minh City Vietnam; ^10^ Institute of Engineering HUTECH University Ho Chi Minh City Vietnam

**Keywords:** acidification potential, ecological evaluation, environmental impact, life cycle assessment, ReCiPe method

## Abstract

Internal combustion engine (ICE) is still the major source of energy for transportation systems worldwide. However, their environmental impacts continue to be a major issue, especially in developing countries like India. Since there is a lack of environmental evaluation of ICE technologies under Indian operating conditions, this study presents a comprehensive Life Cycle Assessment (LCA) of a newly developed diesel engine under Indian operating conditions. The assessment follows the ISO 14040 framework and employs the ReCiPe Life Cycle Impact Assessment (LCIA) methodology across production, operation, and end‐of‐life (EoL) phases. The operational phase is considered the major contributor to environmental degradation, mainly because of the consumption of diesel fuel. When neat diesel is used as the fuel, the highest impact is observed for Climate Change Potential (CCP) with 363,513.60 kg CO_2_‐eq, followed by fossil resource scarcity with 58,358.51 kg oil‐eq and Acidification Potential (AP) with 18,193.93 kg SO_2_‐eq. Photochemical Ozone Formation Potential (POFP) and Fine Particulate Matter Formation (FPMF) also present significant impacts with 259.16 kg NOx‐eq. and 501.45 kg PM‐eq., respectively. The results emphasize the need for new fuel alternatives like biodiesel, technological solutions like hybridization and proper policy measures for successfully reducing environmental emissions associated with ICE.

## Graphical abstract

This study reveals that the use phase of diesel engines contributes the greatest environmental burden due to intensive diesel consumption and related emissions. Life‐cycle assessment highlights severe impacts on climate change, fossil fuel depletion, acidification, and particulate pollution, emphasizing the importance of biodiesel integration, hybrid technologies, and cleaner energy strategies for more sustainable transportation systems.

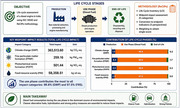

## 1 Introduction

The creation of the internal combustion engine (ICE) is a further turning point in the world of transportation, since nearly all modes of transportation use the propulsive energy from the ICE [1, 2]. Since its innovation, outstanding scientists and academics have made several improvements to increase its use, such as power‐to‐weight ratio, fuel economy, and lower exhaust emissions [3, 4, 5]. Indeed, ICEs have significantly improved fuel efficiency, emissions, and performance due to innovations relating to engine and fuel system design, combustion modes, and alternative fuel technologies [6, 7, 8, 9]. In addition, the use of alternative fuels and additives is considered a useful solution to maximize combustion, minimize emissions and pollution, and improve overall engine efficiency [10, 11, 12, 13]. Nevertheless, the ICE continues to produce pollutants, and the stringent regulations on emissions promote further innovations [14, 15, 16].

Undeniably, ICE is going to survive because of the benefits of infrastructure, long distances, and low costs, although meeting the standards of emissions is still an obstacle. ICE requires higher efficiency and a cut in emissions to be competitive with Battery Electric Vehicles (BEVs). This can be solved by hybridization and plug‐in technology, and cleaner alternative fuels provide a way to mitigate the effects [17, 18, 19]. The replacement of traditional fuels (diesel and petrol) with biodiesel and bioethanol improves the environmental status of ICEs by reducing the occurrence of greenhouse gas (GHG) emissions as well as decreasing the use of fossil fuels [20, 21, 22]. The fact that biodiesel can be mixed with existing diesel engines with few modifications highlights its usefulness [23, 24]. At the core of a car, the ICE consumes many natural resources and manufactures complex and accurate components, which affect the environment. However, there is still a lack of ecological assessment of diesel engine parts in some Asian countries, such as India, Thailand, Vietnam, and Indonesia, where ICE‐based vehicles are being popularly used. Previous studies have mostly examined engine fuel and specific parts, leaving decision‐makers and authorities without enough information to establish industry standards. The automotive sector mostly uses fossil fuels, such as coal, oil, and natural gas, all of which produce significant amounts of GHGs and have a disastrous impact on climate change [25, 26]. Therefore, Life Cycle Assessment (LCA) enables the evaluation of environmental consequences over the complete life cycle of a good or service system, including activities such as material acquisition, manufacture, use, and disposal. An LCA study has four major phases or components, as outlined in the International Standards Organization 14040 series (ISO 2006), including goal and scope definition, life cycle inventory (LCI), life cycle impact assessment (LCIA), and interpretation [27]. Researchers have given attention to performing LCA of ICEs using different conventional fuels (such as gasoline and diesel), and alternative fuels (such as ethanol and biodiesel), and electric vehicles that have much potential to reduce local air pollution.

In the last decade, LCA for diesel engines has been performed for different countries like Sweden, Switzerland, the United States, Canada, Japan, Brazil, China, and Lithuania. According to the results of an LCA research done in Europe, electric vehicles can lower GHG emissions by up to 29% compared to gasoline‐powered vehicles and by roughly 20% compared to diesel‐powered vehicles [28]. An LCA that deliberates upon all these factors is required to provide a thorough LCA computation of ICEs, the energy production mix, and an exhaustive life cycle inventory data system. According to an extensive literature review, the majority of LCA studies on ICE have been performed in China, the United States, Canada, Brazil, and European countries such as Italy. However, there is not enough literature available for Asian countries such as India, Thailand, Vietnam, and Indonesia, so the author is motivated by this gap. Further, a comprehensive assessment of biodiesel's role within ICE is required to pave the way for a greener future in transportation. This endeavour lays the groundwork for the adoption of sustainable practices, guiding us towards environmentally responsible decisions. Our current initiative is embarking on a bold, data‐driven journey based on LCA. The present study aims to investigate environmental impacts through the LCA of an ICE powered by fossil diesel fuel in India, Thailand, Vietnam, and Indonesia. It is worth noting that in the Indian context, the LCA of ICEs powered by fossil diesel fuel is still relatively unexplored. This study aims to fill that gap and contribute valuable insights to the field.

## 2 Literature Review

In the literature, Lior [29] posed that global issues, such as overcrowding, freshwater pollution, air pollution, deforestation, coastal pollution, biodiversity loss, and poor global weather, are swiftly becoming the focus of the energy development process. Mass energy‐related operations would be more incapable of being performed without realizing sustainability to prevent the disastrous international consequences. This holds even in the case of developing countries, where the production and consumption of energy are given more priority than the impacts of such activities on society, the environment, and even the sources of energy. Since ICEs have become so common and replacing them with more sustainable options may be challenging, several barriers can be identified, such as the reduction of the environmental impact of ICEs. The existing infrastructure and dependency on technology are huge challenges [30]. Moreover, Gheewala [31] reported that the crucial component of the comparison is the use of resources. Energy‐intensive procedures are used in the traditional extraction and refinement of fossil fuels, which frequently degrade the environment. However, there are big differences in the way alternative fuels are produced. Land, energy, and water are needed for the production and processing of biofuels.

Guardiola et al. [32] explained that despite the stringent norms of CO_2_ emissions and other pollutants, ICE technology will be able to survive the near future. There are many technologies already equipped and performing well, like EGR, VVT, stop‐start, and many others. Engine control plays an important role at the subsystem level to fulfil the requirement of the exact control of the operations of various advanced technologies. Charge composition and temperature control with different EGR systems, full‐flexible VVT, after‐treatment system control and diagnosis, and system integration are expected to boost engine technology. Alternative fuels like biodiesel are expected to further reduce the emission threat. Alagumalai et al. [33] noticed that the engine industry has experienced tremendous growth in the research and development of new‐age technologies in recent years. Indeed, MPFi, CRDi, GDI, EGR, and low‐temperature combustion modes (HCCI, RCCI, PCCI) are some of the technologies that made remarkable contributions to reducing fuel consumption and pollutant emissions [34, 35, 36]. They further explained that the increasing market for ICE has to be countered by the tailpipe emissions of the engine and the fuel consumption. Due to these two major problems, international pressure to reduce the emissions of GHGs and other environmental pollution, the national government has tightened the emission regulations for ICE [37].

Various developments are necessary to follow these new norms and regulations. However, MPFi, CRDi, and GDI engines are developed to counter various problems of the engines; still, stricter norms are compelling to innovate further to sustain the ICE's future in energy conversion systems. For example, in HCCI engines, more research is required to sort out the lack of stability problem and higher emissions of CO as well as HC. Similarly, lean burn emits more NO_x,_ which needs to be controlled [38, 39]. To enforce biofuel technology systems, optimization techniques, proper planning, and established standards are needed. Although the widespread use of ICEs in various industries highlights their energy density, dependability, and versatility, it also raises concerns about the effects they may have on the environment, notably concerning indoor pollutants and emissions of GHGs. Alternatively, Gheewala [40] in another study explained that a product or system's life cycle consists of several stages, beginning with the extraction of raw materials, which are obtained from nature. Water, energy, and other resources are frequently needed at this stage. The movement of raw materials, components, and completed goods between various places is included in the ensuing transportation phase, which also accounts for the energy use and emissions produced by the various modes of transportation. The product performs as planned during the usage or operation phase, taking into account emissions, energy consumption, and other environmental effects. Throughout the product's life cycle, maintenance and repair tasks can be required, which would affect the product's overall environmental impact. The end‐of‐life phase comprises recycling, management of waste, and disposal, while taking into account the environmental effects of different disposal techniques such as recycling, incineration, and landfilling. Girardi et al. [41] concluded that there are various steps in the LCA process. The first step is to define the aim and scope, which entails stating the purpose of the assessment as well as the boundaries of the system and the particular environmental impact categories that will be assessed. Gathering and quantifying information on inputs and outputs for every life cycle stage is part of the next stage, known as the LCI. Data on resource utilization, emissions, and energy use are included in this. The next step is the LCIA, which uses recognized impact categories like eutrophication, acidification, and global warming to assess the possible environmental effects of the data collected. The last phase, known as interpretation, involves analysing the data to derive conclusions and offer suggestions for enhancement. This process frequently entails contrasting different scenarios or locating “hotspots” in the environment.

In another study by Jiao et al. [42], ICEs have a life cycle that includes multiple crucial stages, all of which have an impact on the ecosystem as a whole. Extracting and processing raw materials, such as metals and other components required for the production of ICE, is the first step in the process. These energy‐intensive procedures, which include mining, refining, and manufacturing, add to the initial environmental impact. The next stage is manufacture and production, when different engine components are made from processed raw materials. This complex process requires a lot of energy, specialised equipment, and resources. It involves casting, machining, heat treatment, and assembly. The construction of ICEs frequently involves various phases and suppliers, resulting in a multitude of components that contribute to its environmental effects. Engine parts are shipped to assembly companies for further processing, including assembly and transportation. Because of fuel use and emissions from moving vehicles, the transportation phase contributes to the environmental footprint of the entire life cycle. Energy and resources are needed for assembly, as parts must be assembled to form the entire ICE. Tribioli et al. [43] described that a significant amount of the ICE's life cycle is made up of the use and operation phase. This phase entails installing the engine and keeping it running in machinery or cars. Air pollution is caused by fuel consumption and emissions during this phase, and the type and quantity of fuel utilized directly affect environmental performance. An ICE may require maintenance and repairs throughout its lifetime to guarantee peak performance. These tasks need more energy and resources, and the final environmental impact can be influenced by the maintenance strategy selected. The ICE retires from active service during the end‐of‐life and disposal stages. There are several different disposal techniques, such as landfilling, recycling, and scrapping. Scrapping is the process of removing reusable parts, whereas recycling attempts to recover precious resources like metals. Environmental issues may arise from improper landfilling, particularly if hazardous materials are not managed properly. In a study by Hawkins et al. [44], a comparative environmental LCA was used to assess the environmental effects of electric vehicles (EVs) with ICE vehicles (ICEVs). The results showed that when EVs were powered by the European energy mix, their global warming potential was reduced by 10–24%. However, issues with EVs’ supply chain have increased toxicity, ecotoxicity, eutrophication, and metal depletion. Variations in impact were associated with assumptions about lifetime, energy consumption, and power supply. The report underscores the need for comprehensive steps for a greener automotive industry future and stresses the need to address production supply chain difficulties and promote clean energy adoption to boost EV sustainability. Kalghatgi et al. [45] expressed concern about the possibility of the disappearance of the ICE‐driven transport system through their study. Any new technology enters the market with the hype cycle, and this is often supported by media houses, generally by focusing only on the positive aspects of a technology. The author suggests that ICE technology has proven itself in the past and can improve by adapting to the changes in the market through new developments and innovations.

The introduction of biofuels like biodiesel and ethanol has a significant capability to counter the environmental emission issue. The production of biofuels will generate an income source for farmers, as well as the utilization of marginal land and wastewater bodies, and the creation of new businesses and job opportunities are additional benefits to the national economy and energy security. The introduction of a fully electrified transportation system has zero tailpipe emissions, but the electricity production is almost coal‐based, which will inversely impact the environment. In addition, various power generation units are not far from the urban areas, which can affect urban air quality. Hence, LCA estimates the consequences of available transport systems in terms of environmental issues to ensure better technology. Indeed, gaps in the literature could be presented as follows:

- The available literature lacks studies utilizing LCA, especially when it comes to thoroughly considering the well‐to‐wheel strategy for evaluating the environmental effects of biodiesel.
- The existing literature lacks comprehensive coverage of the LCA of ICEs, particularly in the Indian setting. This reveals a notable deficiency in the present knowledge base.
- Several investigations show that the ecological advantages of hybrid and electric automobiles are contingent upon the energy mix. However, a more in‐depth examination of the effects of a transitioning electricity system on the LCA of ICEs would yield useful results.
- To comprehend the broader environmental footprint, more research is needed into the cumulative impact of ICE emissions across the whole lifetime, including raw material extraction, transportation, and end‐of‐life disposal.

## 3 Life Cycle Analysis Methodology

The goal and scope of the present work are intended to thoroughly evaluate and enumerate major environmental influences related to the life cycle of diesel engines. The analysis covers the diesel engine's life cycle, including key stages, like transportation of raw materials, extraction of raw materials, component production, engine employment, and end‐of‐life disposal. The major components of the diesel engine are the cylinder head, cylinder block, connecting rod, crankshaft, flywheel housing, timing gearbox, and flywheel, collectively known as the “seven pieces.” These critical components are produced at an engine manufacturing facility in Ghaziabad, India [46]. Data and information from reputable sources, such as the Ecoinvent database and existing literature, are used throughout the LCA process to estimate energy usage and emissions at each stage of the diesel engine's life cycle. Moreover, the research categorizes petroleum, coal, and gas from natural sources as three non‐renewable resources that are crucial for meeting the fundamental energy requirements of the engine's life cycle [47]. The primary objective of this research is to give a complete understanding of the environmental consequences suffered through the diesel engine's entire lifecycle. It hopes to accomplish this by providing critical environmental impact data to decision‐makers in the diesel engine manufacturing industry. It also aims to encourage collaboration with relevant Indian government agencies and to promote the widespread adoption of LCA practices within engine associations. The study aims to drive sustainability practices and reduce the overall environmental footprint of diesel engines in India through these efforts, contributing to a more environmentally conscious and responsible future for the industry.

**FIGURE 1:**
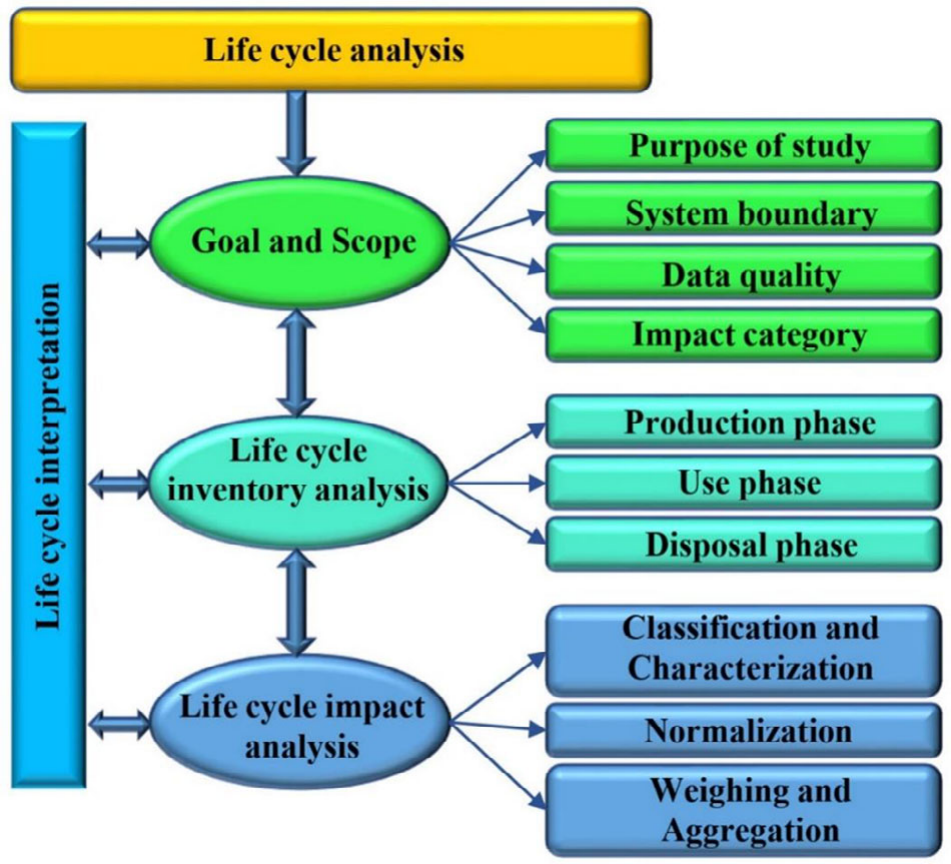
Steps of Life Cycle Analysis.

Evaluating how products and activities affect the environment has become critical in a time of growing environmental concerns and sustainability awareness. A potent methodology known as LCA has been developed to thoroughly evaluate the environmental impact of goods or services throughout their whole life cycle, from raw material extraction to manufacturing, distribution, usage, and final disposal or recycling [48, 49, 50, 51, 52]. The LCA framework, which incorporates best practices through the ISO 14044 and ISO 14040 environmental standards system, has become a global standard for estimating requirements and the effects of processes, technologies, and products. An LCA study is divided into four separate but connected stages [53, 54]. These stages consist of goal and scope, Life Cycle Inventory Analysis, Life Cycle Impact Analysis, and Interpretation of the results. General steps or phases/sub‐phases to accomplish the LCA of a product are depicted graphically in Figure 1 [47, 55, 56].

A methodical Life Cycle Assessment (LCA) employs a structured methodology rooted in the ReCiPe framework, encompassing a systematic development of a life‐cycle inventory and impact evaluation strategy. The principal methodologies employed for this are defined in the subsequent subsections [57, 58].

### 3.1 Life Cycle Inventory Preparation

Total engine operating hours over the lifetime are calculated from daily use and service life [59]:

(1) $$t_{\mathrm{life}}=t_{\mathrm{day}}\;\times d_{\mathrm{year}}\times Y$$

Where: *t*_day_ is the average daily operating time (h/day), *d*_year_ is the number of days per year, and *Y* is the engine lifetime in years.

Fuel consumption over the lifetime is obtained from the measured hourly consumption:

(2) $$V_{\mathrm{diesel},\mathrm{life}}={\dot{V}}_{\mathrm{diesel}}\;\times t_{\mathrm{life}}$$

Where: ${\dot{V}}_{\mathrm{diesel}}$ is the volumetric fuel consumption rate (L/h) and *t*_life_ is the total operating hours. The corresponding fuel mass is:

(3) $$m_{\mathrm{diesel},\mathrm{life}}=\rho_{\mathrm{diesel}}\;\times V_{\mathrm{diesel},\mathrm{life}}$$

with ρ_diesel_ as diesel density.

For each life‑cycle phase *p* (production, use, end‑of‑life) and each emission *i*, the inventory flow is written as:

(4) $$E_{i,p}=\sum_{k}\;a_{i,k,p}$$

Where: *a*_*i*,*k*, *p*_ is the amount of emission *i* from process *k* in phase *p* (e.g., material production, transport, engine operation, recycling). Total life‑cycle inventory for emission *i* is:

(5) $$E_{i,\mathrm{LC}}=\sum_{p}\;E_{i,p}$$

Resource flows (e.g., crude oil, coal, natural gas) are handled analogously:

(6) $$R_{j,\mathrm{LC}}=\sum_{p}\;\sum_{k}\;r_{j,k,p}$$

Where: *r*_*j*,*k*, *p*_ is the number of resources *j* used in the process *k* and phase *p*.

### 3.2 Classification and Midpoint Characterization

After classification, each emission *i* belongs to one or more impact categories *c* (e.g., climate change, fine particulate matter formation, photochemical ozone formation, terrestrial acidification, fossil resource scarcity). The midpoint impact in the category *c* for phase *p* is:

(7) $$I_{c,p}^{\mathrm{mid}}=\sum_{i}\;\left(E_{i,p}\cdot CF_{i,c}\right)$$

Where: CF_*i*,*c*_ is the characterization factor of emission *i* for category *c* (e.g., global warming potential factor for CO_2_, CH_4_, NOx; acidification potential factor for SO_2_, NOx; resource scarcity factors for crude oil, coal, natural gas). The total life‑cycle midpoint impact for the category *c* is then:

(8) $$I_{c,\mathrm{LC}}^{\mathrm{mid}}=\sum_{p}\;I_{c,p}^{\mathrm{mid}}$$

If normalization is applied, each midpoint indicator is converted to a dimensionless normalized value:

(9) $$I_{c}^{\mathrm{norm}}=\frac{I_{c,\mathrm{LC}}^{\mathrm{mid}}}{I_{c}^{\mathrm{ref}}}\;$$

Where: $I_{c}^{\mathrm{ref}}$ is the chosen reference value for the category *c* (e.g., regional or global reference burden).

### 3.3 Endpoint Conversion and Weighting

For each midpoint category *c* and endpoint damage category *d* (human health, ecosystem quality, resource scarcity), ReCiPe provides a midpoint‑to‑endpoint factor ${\mathrm{CF}}_{c\to d}^{\mathrm{EP}}$ for each perspective *v* (individualist, hierarchist, egalitarian). The endpoint impact is:

(10) $$D_{d,v}=\sum_{c}\;\left(I_{c,\mathrm{LC}}^{\mathrm{mid}}\cdot {\mathrm{CF}}_{c\to d,v}^{\mathrm{EP}}\right)$$

This yields, for example, damage to human health in disability‑adjusted life years, ecosystem damage in time‑based units, and resource damage in monetary or equivalent units.

Finally, the endpoint results are aggregated into a single index using the weighting factors *w*_*d*,*v*_ for each damage category *d* and perspective *v*:

(11) $$S_{v}=\sum_{d}\;\left(w_{d,v}\times D_{d,v}\right)$$

Where: *w*_*d*,*v*_ are the weights assigned to human health, ecosystem quality, and resources for the chosen perspective. This scalar score *S_v_* represents the overall endpoint impact of the engine life cycle for that value set and allows direct comparison between phases (production, use, end‑of‑life) and scenarios (e.g., pure diesel vs alternatives).

### 3.4 Functional Unit

Various authors assume that an engine is supposed to be fitted in a car or vehicle; then the distance travelled determines the life of the car, and hence the life of the engine, which ends when the vehicle does. The lifetime of a passenger car is generally taken as 150000 km, and this distance is taken as the functional unit of LCA [41, 60, 61, 62, 63, 64]. However, the present work focuses only on engine manufacturing, operation, and disposal. Also, the engine is assumed to be stationary at the time of operation and used for irrigation purposes. So the functional unit of this LCA is taken as “One Diesel Engine” following the study of Liu et al. [46].

### 3.5 System Boundary

**FIGURE 2:**
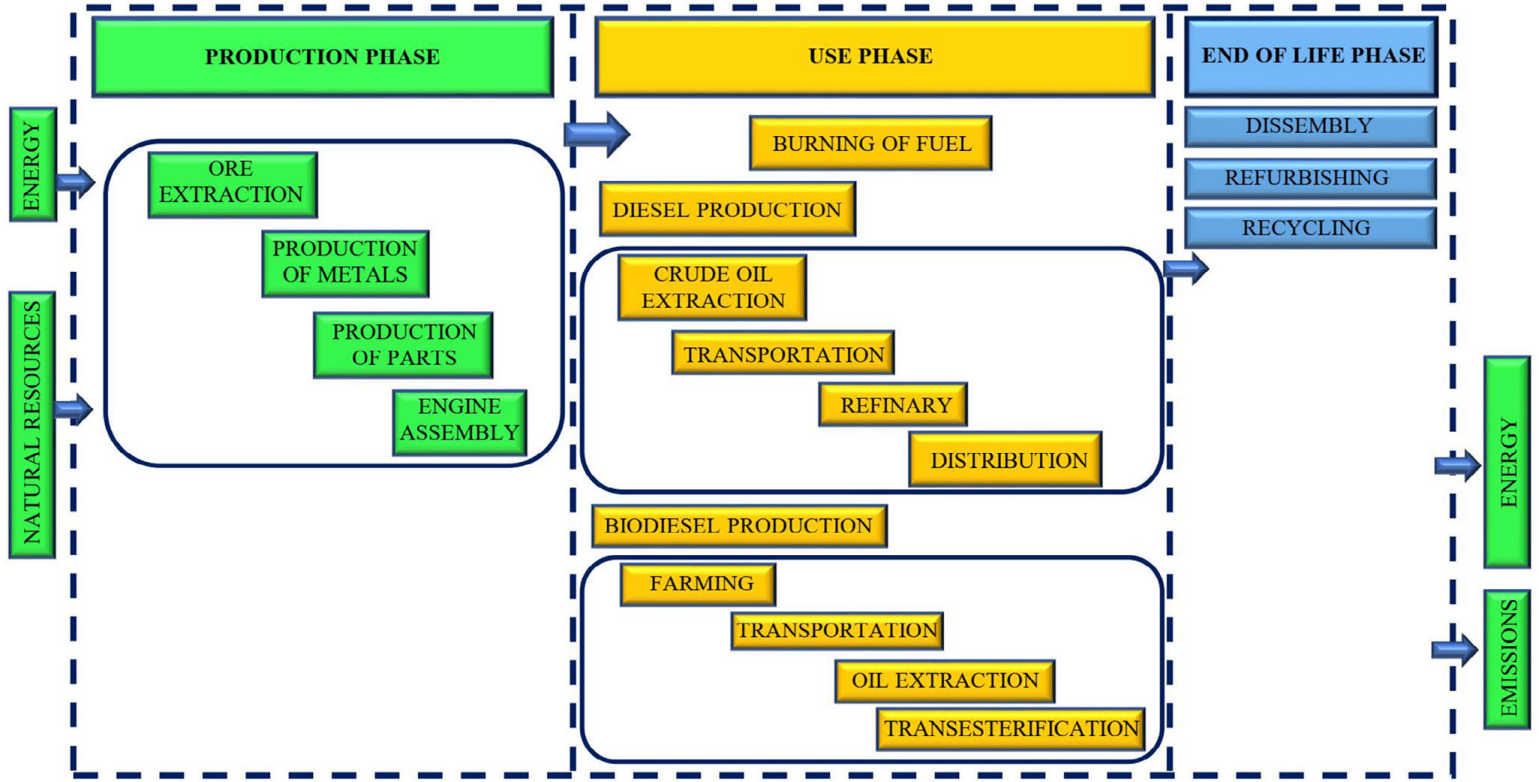
System boundary of ICE.

In this LCA, the “Cradle to Grave” approach was selected, in which the following phases of the system are considered for analysis, including the production of the engine, operational phase of the engine, disposal of the engine, production of diesel, and production of biodiesel. Figure 2 depicts the system boundary of the ICE under consideration in this study.

### 3.6 Environmental Impact

During the life cycle impact assessment (LCIA) phase, different impact categories are evaluated for measuring environmental impacts. The impact factors are selected to ensure that the majority of the major, frequently occurring effects of ICEs in cars have been covered. In Table 1, a few of the impact groups are mentioned. In this work, the guiding principle supplied by the Joint Research Centre in the charter of the European Council on LCA in selecting the methods to evaluate the implications was followed very closely, as available in EPLCA [65].

**TABLE 1:** Environmental impact category.

**Impact Category**	**Reference Substance**	**Reference Indicator**	**Unit of Measure**	**Refs**.
1. Global Warming Potential	CO_2_	IPCC	kgCO_2_ eq.	[66, 67, 68]
2. Photochemical Oxidant Development Potential	NMVOC	ReCiPe	kgNMVOCeq.	
3. Potential of Acidification	SO_2_	CML 2002	KgSO_2_eq	
4. Potential of Particulate Matter	PM10	ReCiPe	gPM10eq	
5. Potential of Eutrophication	PO_4_	CML2002	kgPO_4_eq	
6. Potential of Resource Depletion	Antimony	CML2002	kgSbeq	

The impact groups for the measurement of LCIA are provided by ISO 14040. To make sure that we included most of the key common effects of the dispersion of ICE in the automobiles [56]. Five impact categories were considered for impact assessment, including climate change, terrestrial acidification, fine particulate matter formation, photochemical ozone formation, and fossil resource scarcity. Similar to how GHGs are the main source of temperature rise or global warming, these impacts are the main drivers of environmental changes. Thus, middle‐level indicators are what they are known as. Only certain environmental problems, such as eutrophication, acidification, and climate change, are addressed by midpoint indicators. Endpoint values, on the other hand, show how the environment affects human well‐being, ecosystems, and resource scarcity on three larger levels [41, 46, 69].

## 4 Life Cycle Inventory Analysis

Life cycle inventory is an important phase of LCA. In this phase, the list of all inputs and outputs is prepared. All the items, raw materials, energy resources, and natural resources required throughout the whole life, from their production to the disposal of the product, lie in the input category. While the items produced during the whole life are categorized as output. The life of an ICE can be divided into three major periods for the life cycle perspective according to the cradle‐to‐grave concept of ISO 14040/14044 [27, 70]. The first stage is known as the manufacturing/ production phase, and it begins with the extraction of raw materials and ends with the final construction of the engine, prepared for operation. The second phase is the use phase, where the engine consumes fuel and generates mechanical energy for useful work. As the service life of the engine ends due to the deterioration of its parts and age, the operation of the engine is not beneficial in terms of load, fuel consumption, and environmental emissions. The engine is disposed of at this stage, which is referred to as the end‐of‐life phase or disposal phase. Ecoinvent, GREET database, and literature of reputed journals are used for data requirements [71, 72]. The main outcome of this phase is the cataloguing of different environmental emissions like GHGs, which are used in subsequent phases, i.e., LCIA and interpretation as a groundwork. In this work, all three phases of the engine are studied and given below.

### 4.1 Manufacturing Phase of ICE

There are a huge number of parts made of different materials, including metals and non‐metals, in an ICE. These materials are produced from a particular type of ore, which is extracted from mines and passed through various conversion and refining processes. To obtain the required type of material with the requisite properties to withstand the forces produced in engine operation, various alloying elements are added. These materials are converted into specific parts of different shapes and sizes with adequate strength, as per the design norms, using different manufacturing processes. The required materials to manufacture the parts of an assembly are generally compiled in the form of a bill of materials for that particular product, which is the ICE. A variety of materials is used to manufacture these parts, including steel, cast iron, aluminium, copper, and plastic. It is impractical to consider all parts of the ICE for LCA. Therefore, major parts are considered for this LCA, and there are seven major parts of the ICE, which include the cylinder, cylinder head, piston, crankshaft, and flywheel, which constitute over 98% of the total weight of the engine [46]. In the selected engine, it was found that steel, aluminium, cast iron, and alloy are mainly used to produce these parts and weigh about 260.50 kg, which is about 97.6% of the total weight of the engine materials (267.00 kg). Other components, like rubber and a tiny quantity of polymeric substances, are omitted since the resources and energy used in creating these materials may be believed to be minimal, or the emission outcomes can be included with a reasonable proportion.

Smith et al. [73] conducted a thorough investigation into the energy consumption associated with the production of diesel engines within an engine manufacturing plant. Their findings revealed that the total energy consumption for manufacturing a diesel engine, including materials procurement, part production, and assembly, was 11,600 MJ. A more practical approach was adopted in another study to evaluate energy consumption and, hence, the environmental emissions by considering the manufacturing of only critical components. The critical components considered were the cylinder head, cylinder block, connecting rod, crankshaft, gearbox, as well as the flywheel and flywheel shell, which are referred to collectively as the “seven pieces.” This estimate is carefully carried out by calculating the realistic power needs of every piece of equipment based on its machining time. Furthermore, the energy is used in the manufacture and assembly of other necessary accessories such as bolts, belt pulleys, and water pumps. The research relies on the concept of “experience‐specific energy” derived from various manufacturing processes to estimate these energy expenditures [74].

In the present work, it was assumed that the parts of the engine under consideration were produced in Ghaziabad, Delhi National Capital Region (India), and the engine assembly was also completed within this plant. For the manufacturing of the IC engine, recyclable materials are also used; however, the percentage of recycled material in each category is unclear, and varies over time depending on scrap supplies. As a result, no recycled material is taken into account in the model; instead, only the usage of primary or virgin material is modelled for the baseline. EcoInvent is a database that is commonly used for data requirements in LCA and contains data on the number of materials that constitute each of the parts of an ICE. Due to the lack of India‐specific EcoInvent data, the total weight of each material component was calculated using the worldwide average value. As the EcoInvent database lacks information on the transmission of combustion vehicles, data about the transmission of ICEVs was gathered from Sullivan et al. [75].

**FIGURE 3:**
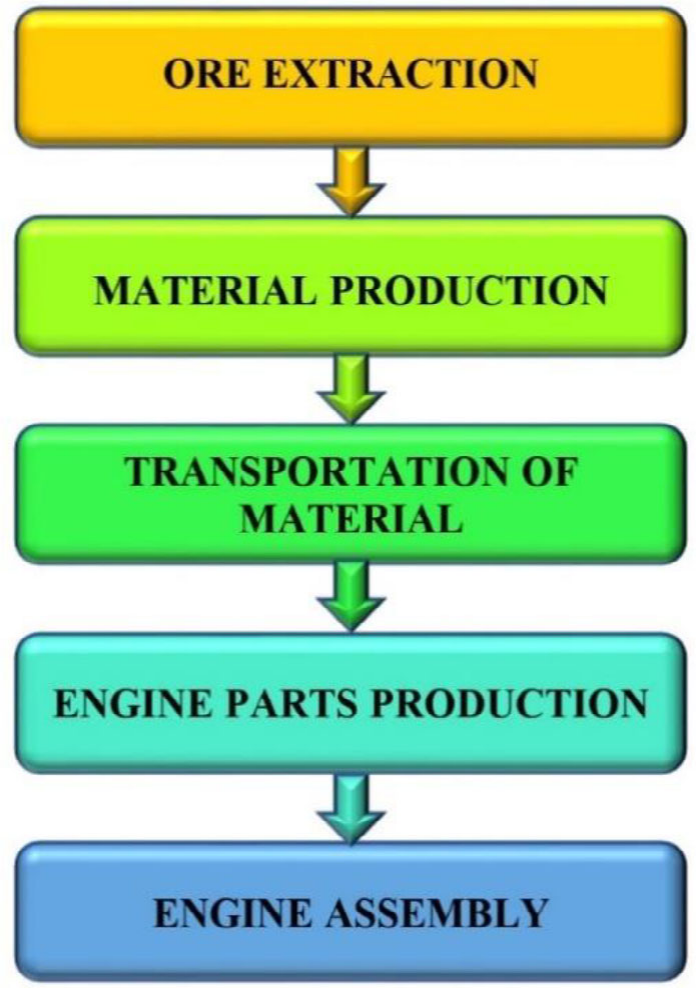
Main steps in ICE production.

The data on embodied energy were acquired from the Indian Construction Materials Database of Embodied Energy and Global Warming Potential, which was released by the *International Finance Corporation of the World Bank* in 2017. This database contains information on the carbon dioxide equivalent emissions generated during the manufacturing process of materials, including steel, aluminium, and glass [76]. The energy and GHG emissions associated with the materials were calculated by multiplying the amount of each substance in each component. This aligns with the Make in India program of the Government of India [77]. EcoInvent incorporates the quantity of electrical energy used in the extraction and making of materials, as well as the manufacturing of components of the engine, and the subsequent assembly of these components to obtain the final shape of the engine. To determine the amount of GHG emissions associated with the energy utilized in the automobile manufacturing process, the amount of electrical energy has been multiplied by the carbon footprint of the grid for each year. The flowchart of the production steps of the ICE is shown in Figure 3.

#### 4.1.1 Extraction and Refining of Materials Production

Upon dismantling the ICE and identifying the materials used for making its constituents, it can be concluded that most of the parts are made of mainly three materials, i.e., cast iron, steel, and aluminium [72, 78]. Hence, the material production part of this study is limited to the production of these materials only. Iron ore obtained from the above methods is separated from other minerals using magnetic or gravity separation techniques, known as beneficiation. The extraction of iron from its ore is accomplished by heating the iron ore with carbon in a blast furnace, which produces the pig iron in molten form. This pig iron can be further used to produce cast iron or steel as per the requirement [79]. Each of these systems has distinct benefits and drawbacks in relation to expenses, effectiveness, ecological consequences, and security. The choice of technique used is contingent upon variables such as the geographical placement and magnitude of the iron ore reservoir, the calibre and constitution of the ore, and the accessibility to assets and technology. In terms of environmental impact, the best method for extracting iron ore is open‐pit mining if it is done in an environmentally responsible manner. This is because open‐pit mining has a smaller footprint compared to underground mining, and it is less likely to cause significant damage to the surrounding ecosystems. Open‐pit mining also has a lower energy requirement, which means fewer GHG emissions and less air pollution. As a result of the present study, open‐pit mining is assumed for iron ore mining [80].

The extraction of iron ore involves several processes that are responsible for emitting various types of pollutants into the environment. Main pollutants in iron ore mining are identified as CO_2_, SO_2_, NO_x_, CO, PM, sulfuric acid (H_2_SO_4_), methane (CH_4_), volatile organic compounds, and heavy metals. The GREET database of global types and other research studies are used to compile the data for emissions in the mining of iron ore. The National Mineral Development Corporation (NMDC), with an output of 35.57 million tonnes in 2017–18, has the distinction of being India's biggest iron ore mining corporation. The 2018 sustainability report of NMDC provides specific information on the amount of fuel and energy used in its mining operations [81]. The input inventory in the mining operation of iron and bauxite ore is given in Table 2.

**TABLE 2:** Input for iron and bauxite mining.

**Name of the item**	**Required for Iron ore**	**Required for Bauxite (Al ore)**	**Refs**.
Diesel (Kg/Ton Ore)	3.4	.93	[82, 83]
Electricity (KWh/Ton Ore)	3.8	2.0	
Explosive (Kg/Ton Ore)	0.5	0.3	
Water (m3/Ton ore)	0.21	0.3	
Total Energy (MJ/Ton)	175	70	

**FIGURE 4:**
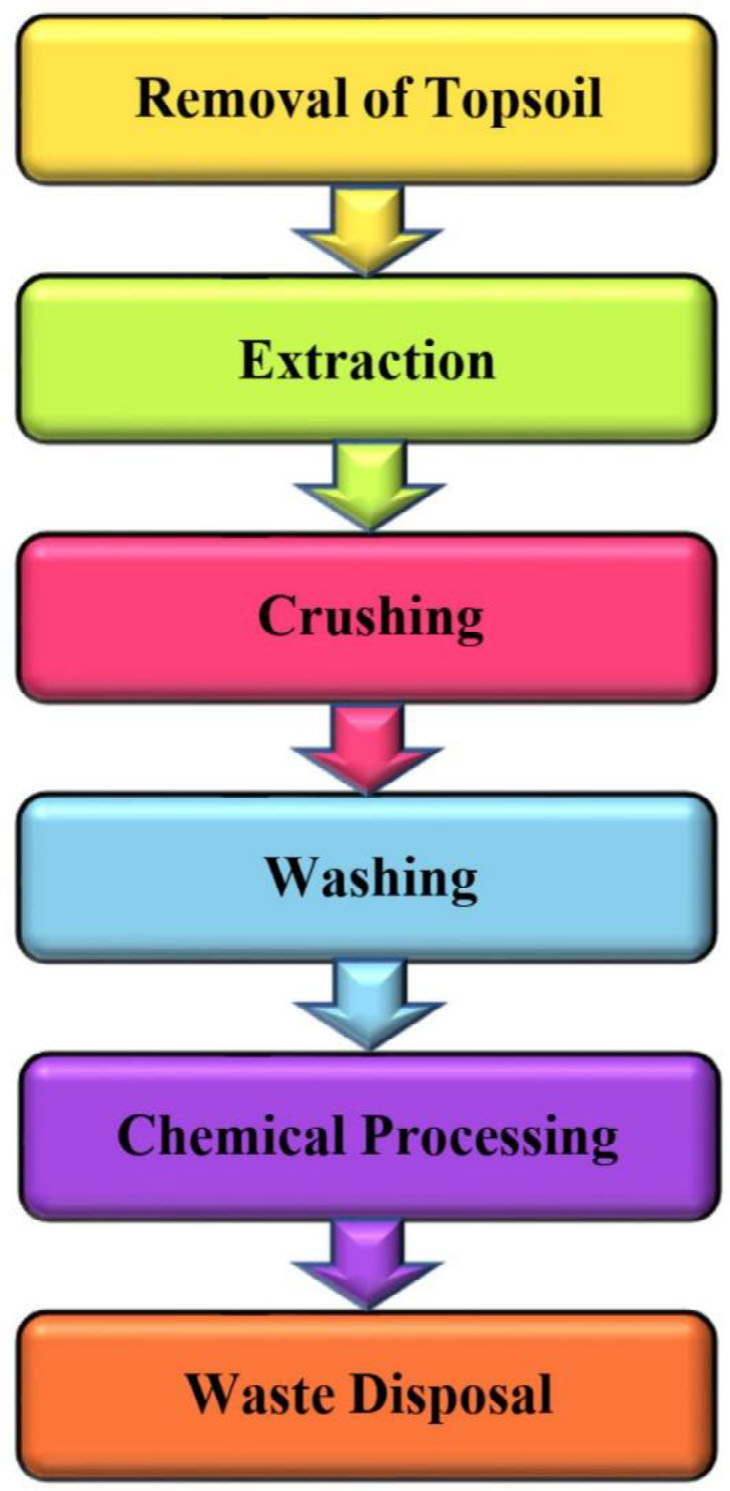
Bauxite mining.

Bauxite is the main ore from which aluminium metal is produced. The capacity of bauxite production in India increased in 2019, reaching 23.2 Mt compared with 22.3 Mt in 2018 [84]. Bauxite is typically mined using open‐pit mining methods, which begin with the removal of overburden to access and extract the bauxite ore beneath. The extraction process involves the use of heavy machinery, such as excavators, trucks, and conveyors, all of which consume fossil diesel and pollute the environment significantly [85]. The major steps are depicted in Figure 4.

To remove impurities, the extraction process is followed by crushing and washing. This process necessitates the use of large amounts of water, which can deplete local water supplies and pollute groundwater with sediment and chemicals used in the washing process. Waste disposal is also an important aspect of bauxite mining. Heavy machinery, such as bulldozers, excavators, and trucks, can cause soil erosion, habitat destruction, and water source disruption [86]. After the bauxite ore is extracted, it is transported to a processing plant where it is crushed and washed to remove impurities [87]. The crushed and washed bauxite ore is then subjected to chemical processing to extract aluminium oxide, also known as alumina, through a process called the Bayer process. This involves the use of caustic soda (sodium hydroxide) and other chemicals, which can result in the generation of toxic sludge and the release of GHGs such as carbon dioxide [88].

The waste generated during the bauxite mining and processing process, including overburden, sludge, and other waste materials, needs to be disposed of properly; otherwise, the contamination of soil, water, and air can have long‐term negative impacts on the environment and local communities [87]. Bauxite mining has significant environmental impacts, including deforestation, loss of biodiversity, soil erosion, water pollution, air pollution, displacement of local communities, and deficient rehabilitation and restoration efforts. In the processing and washing of extracted bauxite, large amounts of water are used [89]. The runoff can contain toxic substances, such as heavy metals and chemicals used in the mining process, which can contaminate nearby water sources, including rivers, lakes, and groundwater, leading to water pollution. Water pollution can have detrimental effects on aquatic ecosystems, affecting fish and other aquatic organisms, as well as human health if contaminated water is used for drinking or irrigation.

The utilization of heavy machinery, such as bulldozers, excavators, and trucks, which emit dust and particulate matter into the air, is a major source of air pollution [90]. Dust and particulate matter can settle on vegetation, soil, and water bodies, leading to air pollution. The release of GHGs during the extraction and transportation of bauxite can also contribute to climate change. According to a report prepared by PE Americas, 5246 kg of bauxite is required to produce one tonne of primary aluminium. It yields 1915 kg of alumina, and after electrolysis reduction, approximately 1018 kg of liquid metal is obtained, followed by the preparation of 1000 kg of ingot and the recycling of the remaining metal [87]. The input and output of materials, energy, and emissions for bauxite mining in this study were also derived from the GABi and GRRET report, which used a global average.

**FIGURE 5:**
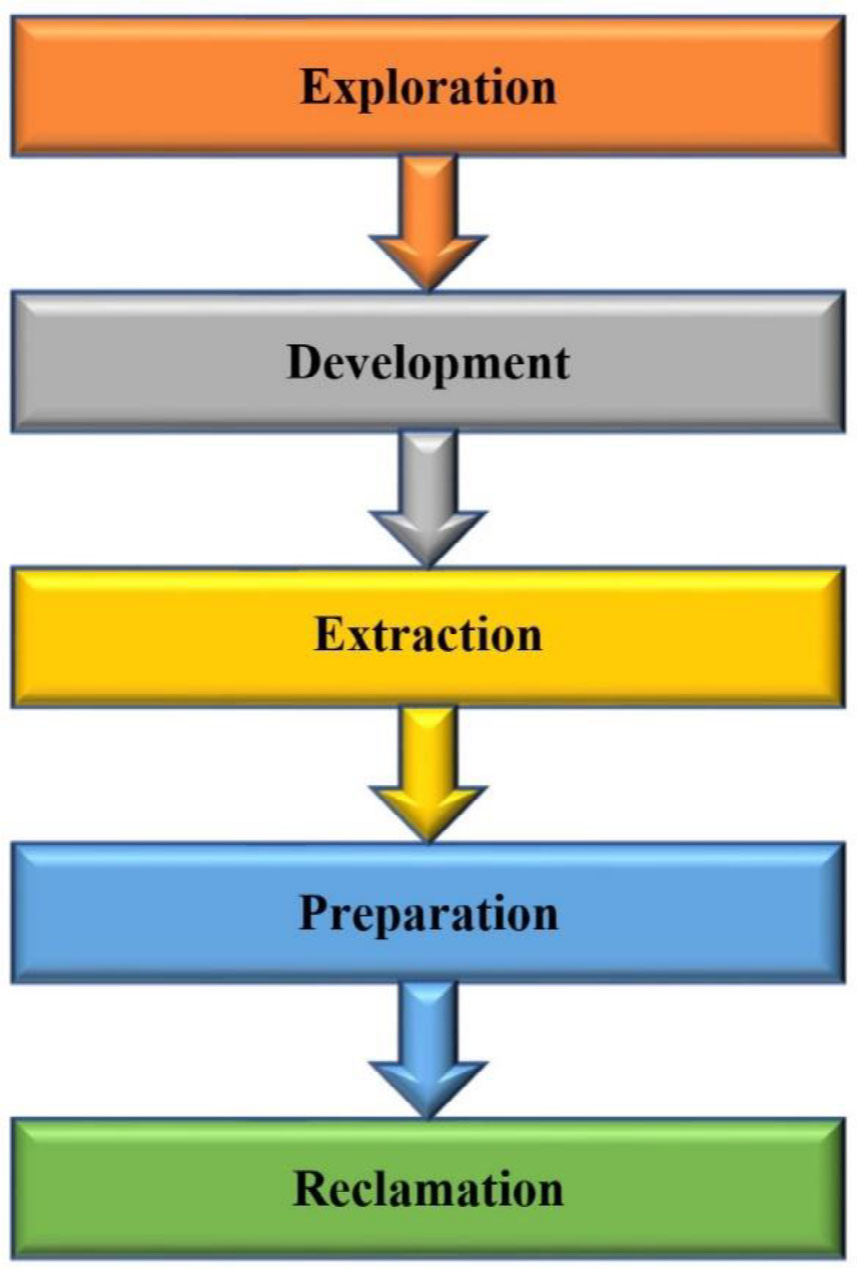
Steps of coal mining.

In India, the combustion of coal is responsible for two‐thirds of the total CO_2_ emissions in the country. Nonetheless, fugitive emissions from coal mining activities contribute significantly to global GHG emissions. Based on the findings of top‐down modelling, it is evident that even with a robust 2°C transition route, these emissions will continue to be substantial until the conclusion of the century, amounting to around 300 Mt‐CO_2_e. Consequently, it is crucial for India, now the second‐largest coal producer globally, to engage in an accurate and comprehensive assessment and comprehension of mitigation strategies. The sustainability report released by CIL in 2016–17 states that diesel use is 0.79 litres per tonne of coal or 8.12 kilowatt‐hours per tonne of coal. Additionally, the electricity consumption in mine operations is 8.82 kilowatt‐hours per tonne of coal. These particular consumption figures are estimated based on the coal output in 2016–17 [91]. Major inputs for coal mining are water (69.05 Litters per ton of coal), diesel (1.732 Litres/ton), electricity (0.709 kWh/ton), and explosives (0.13 Kg/ton of coal) [92]. In general, the coal mining steps could be depicted in Figure 5.

#### 4.1.2 Production of Engine Parts

It was assumed for this research that the engine parts in question were produced in Ghaziabad, and the engine assembly process took place entirely at this location. The ICEs were made using recyclable materials, but the precise proportion of recycled material in each category was not fixed and changed over time as a result of changes in the availability of scrap. As a result, the model only focused on employing primary or virgin materials as the baseline and ignored recycled resources. Following the study by Liu et al. [46], the manufacturing processes for the engine block, cylinder head, crankshaft, connecting rod, flywheel, crankcase, piston, camshaft, and inlet/outlet manifold were studied for manufacturing purposes. The engine block is the main and quite stressed structure that is fitted with several important parts, such as the crankshaft, camshaft, cylinder heads, pistons, cylinder liners, crosshead, fuel injection pumps, governor, and turbocharger support, to form a whole powerpack. The production sequence of a cast iron engine block involves casting a rough structure and then performing machining operations to give it its exact shape. Generally, a cupola and an induction furnace are used to melt the metal at about 1500°C. Pig iron, steel scrap, and ferrosilicon are used as the main raw materials. For the melting operation, about 4GJ/ton of energy is required. The melted metal is held in a holding furnace, from which it is sent to the molds as per the requirements and production rates. The energy requirement for holding a furnace is about 0.2 GJ per ton of metal. Metal losses are also noticed at the rate of 2% in this stage. Cores are used to provide the internal complex shape of the block, which also consumes energy for the coating and baking. About 0.97 GJ of energy per ton of core sand is consumed in core making. Moulds are prepared by machining and used to provide the outer surface of the part, which also consumes energy at the rate of about 0.2 GJ per ton of green sand. Generally, gravity sand casting is used to cast the engine block. After solidification, fettling is used to remove the part, and rough machining is used to remove the risers, gates, runners, and secondary cavities. Finally, about 1GJ per ton of metal is consumed in this process [78, 93, 94, 95].

The cylinder head is secured to the cylinder block with studs or bolts. Cylinder head manufacturing includes blank forging followed by machining. In a study, gravity casting with metal molds was applied, in which a 5‐ton capacity induction furnace was used for metal melting at 1580°C. The electricity consumption was 112.75 kWh. In machining, the cause of environmental pollution is the consumption of electricity, and the electricity consumption for machining a cylinder head is 189.23 kWh. Therefore, “Indian Grid Power Mix” is used to calculate the environmental impacts of the consumed electricity [96, 97].

The engine's crankshaft is possibly the single most expensive component in a diesel engine. It is the medium that converts reciprocating motion into rotational motion. The forging process is used to fabricate the crankshaft, aiming to ensure that the grain remains continuous. For the forging, the billet is initially heated in a heating oven whose cycle time is 15 mins, and then forged with a hydraulic press of 1000 T, which also takes 15 mins. The forged crankshaft is machined with a CNC turning machine, which takes 70 mins [98, 99, 100].

The connecting rod is a part that connects the piston to the crankshaft and serves as a medium for transforming reciprocating motion into rotational motion. The connecting rod's length is normally between 4 and 5 times the crank radius. They are I‐beams made of fine‐grained, totally killed alloy steel forging. Connecting rods feature a fine‐drilled hole from the large end to the small end, allowing oil to lubricate the small end bearing and piston pin, as well as to cool the piston. Initially, the billet is heated to forging temperature in the heating oven of Banyard for 15 mins and then processed in a hydraulic press of 250 T with a cycle time of 10 mins. A pneumatic press of 150 T is used for trimming purposes, and it takes 5 mins. A CNC turning machine is used for machining operations, which are completed in 25 mins [101, 102, 103].

The flywheel accumulates energy from power strokes and smoothly transmits it to the vehicle's drive train, which connects the engine and gearbox. The flywheel of big, low‐speed engines is generally composed of cast iron. The FG Iron Casting Jolt squeeze sand molding machine, model MAKE‐DISA 1300, is used to obtain the raw casting of the flywheel. The cycle time is 3minutes along with the Shell core shooter machine. Qingdao Antai AT958 is used for core preparation, having a cycle time of 5 mins. After obtaining the casting, machining and tooth cutting are done by the CNC turning 2000 with a 40‐minute cycle time, and gear shaping with a 90‐minute cycle time [104].

The crankcase is a major part of an engine that holds the crankshaft in place for its rotation. It is generally made of cast steel. It also provides the facility of an engine foundation [93, 105]. The FG iron casting jolt squeeze sand moulding machine, made by DISA 1300, is used for casting, which has a cycle time of 3 mins. The shell core shooter machine Qingdao Antai AT958 is used for core making, having a cycle time of 5 mins. The cast structure is processed for necessary machining operations using a horizontal machining centre, which takes 60 mins.

The piston is an essential component in the diesel engine as it serves as a constituent of the combustion chamber and actively contributes to the power transmission process. The burning of fuel generates a substantial quantity of heat. It is made of aluminium alloy. A die‐casting machine is used to produce the raw blank, and one piece is produced in 15 min. The blank is processed using a CNC turning machine, which takes 25min. Final finishing is performed using cylindrical grinding and Jig grinding, consuming 15 min and 10 min, respectively [93, 106].

The camshaft in diesel engines plays a crucial function in controlling the opening and closing of the intake and exhaust valves. Typically, the camshaft is made up of alloy steel. A raw billet is heated in an oven for 15 min and then processed for the forging operation using the hydraulic press of 1000 tons, which takes 10 min. A CNC turning machine is used for machining operations, and it takes 15 min. After that, the profile grinding machine performs the final finishing operations in 20 min [107].

The design of the intake manifold should prioritize minimizing sidewall friction and ensuring a lower temperature to prevent charge re‐ignition. Novel composites are suggested to save production expenses and enhance thermal efficiency [108]. There are more components like nuts and bolts, rocker arms, pins, and diesel tanks, which are not described in this work. However, the energy consumption and hence the consequence of environmental emissions for these parts' manufacturing are assessed through the “experience‐specific energy” approach of different manufacturing processes [74].

#### 4.1.3 Assembly of ICE

It was assumed that the engine was produced by assembling the various components in the factory manually. Power‐operated tools and equipment are used to perform various assembly operations, like nut and bolt tightening, and transportation of heavy parts, such as crankcases or flywheels, from the store to the assembly floor. Electricity consumption is a major cause of environmental pollution in assembling the engine. The energy consumed in the assembly of a six‐cylinder diesel engine is reported in a study by Liu et al. [46]; the energy consumption is thus calculated proportionately [46].

### 4.2 Use of Diesel Engine

It was presumed that an ICE is used for 20 h each day, specifically for agricultural irrigation, and the fuel consumption for one hour is noted at various time intervals and different loading (also, a survey is performed with farmers of the rural area).

Diesel engine life: 20 years = 20*365 days = 7300 days = 7300*10 hrs (assuming that an engine runs 10 hrs/day) = 73000 hrs

Amount of diesel consume per hour = 800 mL = 0.8L (by experiment)

Amount of diesel consumed in 20 years (73000 h) = 0.8 × 73000 L = 58400 L

Mass of diesel consumed in 20 years (73000 h) = 0.85 × 58400 L = 49640 kg

The production of diesel, including the steps of crude oil extraction, transportation, and the refinery process to obtain diesel suitable for engine combustion, was considered in the usage phase, along with the consumption of diesel.

### 4.3 End‐of‐Life Disposal

Out‐of‐service diesel engines will be gathered and returned (via reverse logistics) to the engine production factory in Ghaziabad. Engine components are detached and cleaned, followed by inspection to further work on them whether to refurbish the individual part for reassembly or recycle it. It is observed that about 85% of the engine parts are satisfactorily reused, and only refurbishing is required for further use in the engine. These components have more than 90% of the total weight of the engine [109]. Energy is consumed in the above operations, like the separation of parts, washing/cleaning, checking, and refurbishing, and again assembling the engine. Other components that are not in a condition for reuse, even after the refurbishing process, are sent for recycling. A furnace of 600kw capacity suitable for metal melting is used for recycling the leftover components. Power consumption for melting cast iron, steel, and aluminium is 560kwh, 600kwh, and 400kwh, respectively. Final electricity consumption is estimated by multiplying the weight of the metal [46]. In general, major emissions and utilization of natural resources for all three phases are listed in Tables 3 and 4.

**TABLE 3:** Emission inventory of the diesel engine life cycle.

**Production Phase**								**Use Phase**			**End of Life Phase**			**Refs**.
**Environmental emission**	**Production of materials (kg)**				**Material transport**	**Engine manufacturing**	**Total emission of** **engine production**	**Pure diesel case**			**Components refurbishing**	**Materials recycling**	**Total**	
	**Cast iron**	**Steel**	**Aluminium**	**Alloy**				**Production**	**Combustion**	**Total emission in use phase for pure diesel**				
CO_2_	418.74	139.3	294.35	19.32	18.55	1085.93	**1976.19**	18618.51	157014	**175632.51**	315.18	28.98	**344.16**	[31, 46, 72, 74, 86, 95, 104, 107, 110, 111, 112, 113, 114]
CO	0.09	0.033	0.06	0.32	0.168	0.24	**0.92**	21.62	547.2	**568.2**	2.78	0.26	**3.04**	
CH_4_	1.03	0.31	0.83	0.058	0.0009	3.21	**5.44**	1019.44	2.94	**1022.38**	1.12	0.11	**1.23**	
NO_X_	0.45	0.18	0.73	0.058	0.11	3.13	**4.66**	31.07	464.37	**495.44**	1.22	0.12	**1.34**	
SO_2_	0.89	0.37	1.02	0.048	0.05	3.79	**6.16**	131.54	4.97	**136.51**	3.18	0.29	**3.47**	
PM	2.52	0.87	0.58	0.12	0.004	14.69	**18.78**	104.08	34.75	**138.83**	2.95	0.27	3.22	
H_2_S	0.0021	0.0006	0.0062	0.0003	0.00005	0.00	**0.01**	0.24	0	**0.24**	0.14	0.02	0.16	
HCL	9.90E‐03	7.30E‐03	6.30E‐02	2.00E‐03	2.50E‐04	0.31	**0.39**	1.52	0.00	**1.52**	0.08	7.20E‐03	7.28E‐03	
VOC	0	0	0	0	0.003	0	**0.00**	**0**	0	0	**0**	0	0	
BOD	1.09	0.384	0.128	0.01	0.0052	0.01375	**1.64**	366.02	0	**366.02**	0.25	0.023	0.273	
COD	1.135	0.41	0.194	0.06	0.0075	0.02375	**1.83**	431.27	0	**431.27**	0.29	0.027	0.317	
NH_4_	0.0057	0.0015	0.0023	9.4E‐05	0.0037	0.0004875	**0.014**	10.39	0	**10.39**	0.003	0.0003	0.0033	

**TABLE 4:** Natural resources inventory in the diesel engine life cycle [29, 46, 86, 94, 106, 115, 116, 117].

**Natural resources utilization**													
**Production phase**								**Use phase**			**End of life phase**		
	**Production of materials (kg)**				**Material transport**	**Engine manufacturing**	**Total**	**Pure diesel case**			**Components refurbishing**	**Materials recycling**	**Total**
	**Cast Iron**	**Steel**	**Aluminium**	**Alloy**				**Production**	**Combustion**	**Total**			
Crude oil	204.91	57.34	159.54	11.53	2.38	697.29	**1132.99**	4429.47	0	**4429.47**	236.21	15.5101	**251.72**
Coal	8.87	3.02	6.53	0.69	29.57	4.16	**52.84**	62455.42	0	**62455.42**	27.36	1.7974	**29.16**
NG	0.21	0.74	2.15	0.82	0.48	6.2088	**10.61**	30.08	0	**30.08**	2.22	0.1462	**2.37**

## 5 Results and Discussion

### 5.1 Life Cycle Assessment Inventory Analysis

**FIGURE 6:**
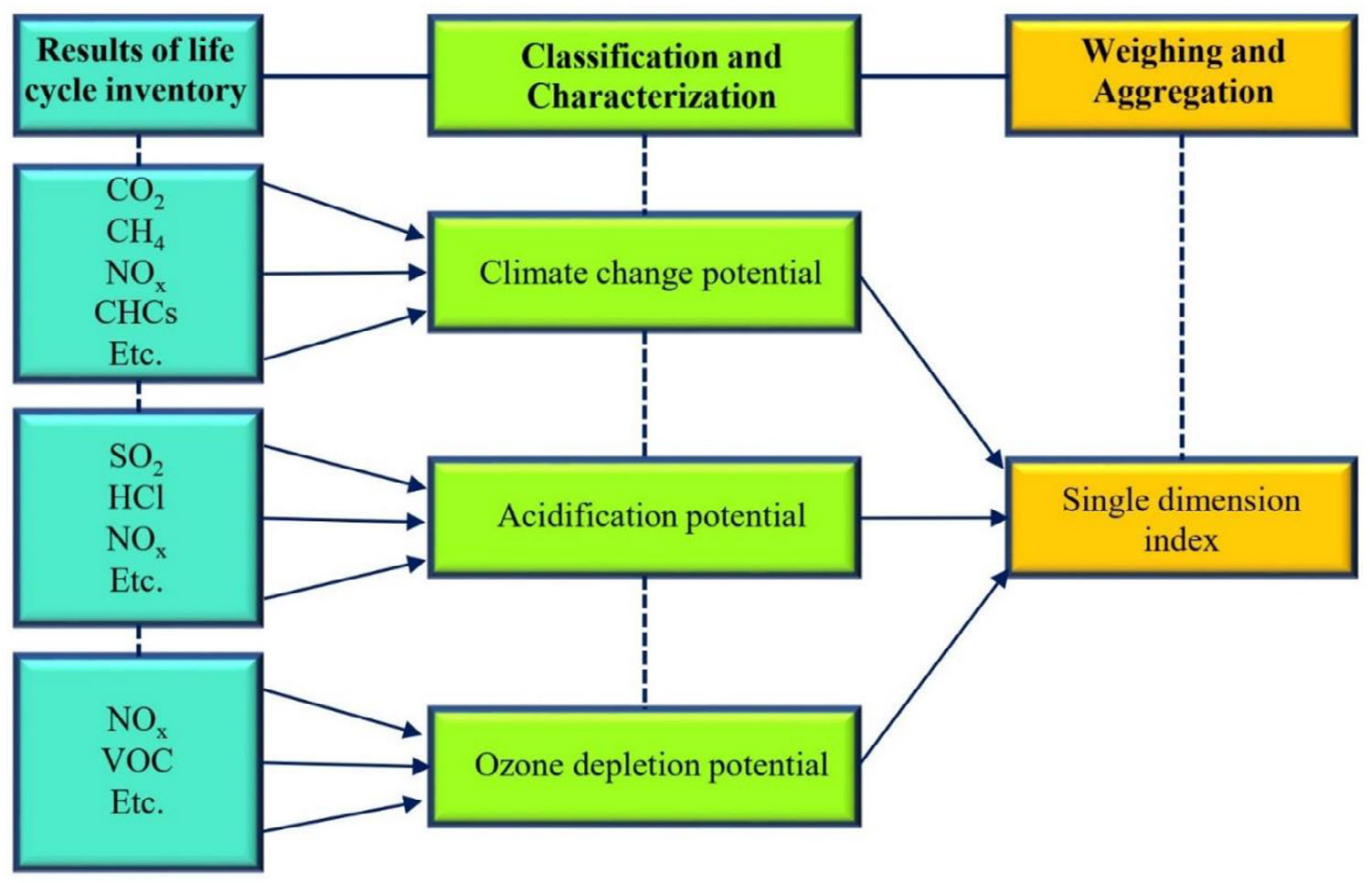
General procedure of Life Cycle Impact Assessment.

The Life Cycle Assessment (LCA) inventory analysis offers the standards by which LCIA approaches weigh the different environmental effect categories. Environmental emissions from the life cycle inventory phase, such as CO_2_, SO_2_, NO_X_, COD, BOD, and HCL, are utilised for determining the influence on the environment and subsequently on humans or other living things [118], as shown in Figure 6. The LCIA step involves the association of LCI results with environmental effect indicators and categories. This is accomplished using LCIA techniques, which first divide emissions into impact categories and then characterise them using characterization factors to enable comparison. It is the phase of life cycle assessment involving the compilation and quantification of inputs and outputs for a given product system throughout its life cycle. The third stage of an LCA focuses on comprehending and assessing the possible environmental effects of the product system or systems under investigation, including their scale and relevance [65]. ISO 14040 provides guidelines to perform the LCIA, which generally includes classification, characterization, and normalization [27].

Emissions come in many different forms, including CO_2_, CH_4_, NOx, and others, which can have multiple negative effects on the ecosystem. As a result, each emission collected during the inventory phase is categorised based on its impact category. This process is referred to as classification for life cycle impact assessment. The characterization process is the calculation of an impact category quantitatively due to a certain type of emission substance. This is achieved by the multiplication of the emission quantity by the characterization factor [27]. The purpose of normalisation and weighing is linked to the purpose and scope of the study. It therefore depends on the quantity and type of alternatives and impacts included, as well as the system boundaries and intended audience. Normalization is not mandatory but optional according to ISO 14040. By responding to the question of whether the results’ order of magnitude is believable, normalisation can be very helpful in guiding the interpretation stage of LCA. In order to help with the interpretation and dissemination of the impact results, it can also be utilised to compare the outcomes with a reference scenario that is separate from or unrelated to the case studies [119]. There are various methods developed for life cycle impact assessment by different agencies from time to time. According to the ecoinvent database, a few of the techniques are listed below [113], including CML 2001, Eco‐indicator 99, EDIP—Environmental Design of Industrial Products 2003, Ecological Footprint, IMPACT 2002+, ReCipe, Ecological Scarcity 2006, IPCC 2001 (Global Warming), IPCC 2007 (Global Warming), and Ecological Scarcity 1997. A few of the techniques exclusively define environmental emissions at one level, also referred to as the midpoint level indicator. The midpoint level indicator conveys the effects of environmental changes, such as rising temperatures. Other approaches divide the environmental emissions into two categories: endpoint and midpoint. Three primary kinds of endpoint‐level indicators are listed as human health, ecosystem quality, and resource scarcity [67].

**FIGURE 7:**
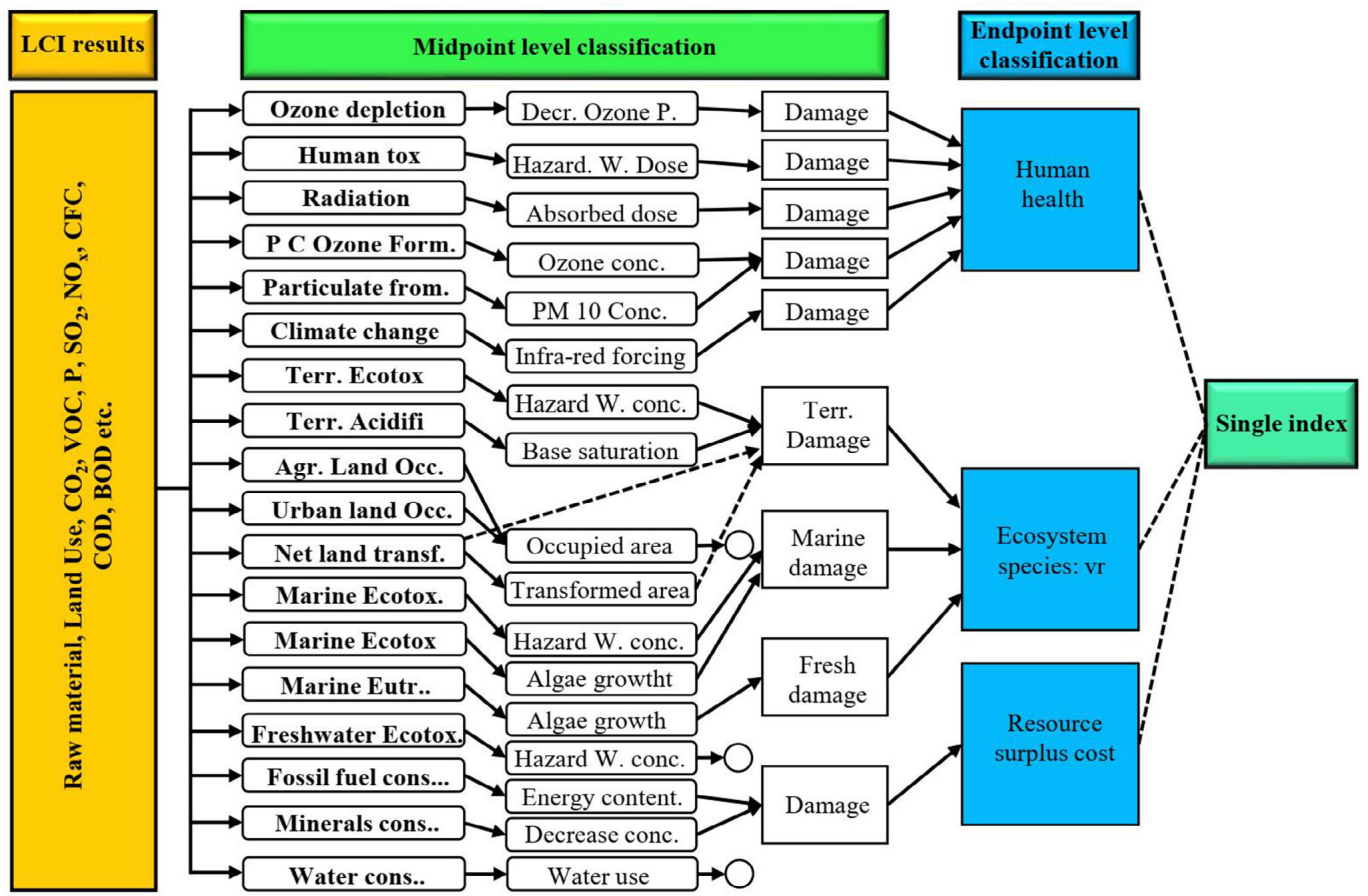
Steps of the ReCiPe impact method.

The endpoint level indicators are further computed on a temporal basis, based on the three perspectives. These three viewpoints are egalitarian (for an extended period of up to 1000 years), hierarchist (for a medium period of up to 100 years), and individualist (for a short period of up to 20 years). The ReCiPe method is used, offering midpoint and endpoint indicators. When compared to methods that focus solely on one approach, ReCiPe stands out as a versatile tool that caters to a broader range of applications. It provides detailed data at the midpoint level for precise analysis as well as user‐friendly endpoint information for the average person [54]. Despite its goal of impartiality and offering a variety of ideological options, the endpoint weighting in ReCiPe may be perceived as a bit random. Interpreting LCAs aids in regulatory compliance and risk management by informing resource allocation decisions [53]. It boosts stakeholder engagement and encourages continuous improvement. Overall, it enables decision‐makers to prioritise safety, health, and sustainability while dealing with complex environmental issues. The representation of the ReCiPe impact method is shown in Figure 7.

### 5.2 Classification and Characterization at the Midpoint Level

The emissions obtained in the second step of LCA, i.e., lifecycle inventory analysis, are classified in the lifecycle impact assessment. Following the ReCiPe, the operation is carried out. To get the midpoint level indicator, multiply the value for a particular type of emission derived from the life cycle inventory by the characterisation factor. Table 5 provides information on the study's environmental impact at the midpoint indicator [66]. It shows the factors that have been used to classify and characterize the life cycle inventory (LCI) emissions to midpoint environmental impact categories in the ReCiPe life cycle impact assessment framework. The characterization factors are used to quantify the relative contribution of each pollutant to a specific environmental impact category. The reference substance for climate change potential (GWP) is CO_2_ with a characterization factor of 1kgCO_2_‐eq/kg emission. Other pollutants have much higher global warming potentials, with CH_4_ at 26.2, CO at 5.53, and NOx at 367 kgCO_2_‐eq, showing that they have a significant effect on climate change on a mass‐emissions basis. Particulate matter (PM) has the highest impact factor of 4.99 kgPM10‐eq, followed by SO_2_ (1.26), NOx (0.47) and NH_3_ (0.26) in the fine particulate matter formation category, highlighting their contribution to degrading air quality and human health risk. NOx has a higher characterization factor of 1kgNMVOC‐eq for the formation of photochemical O_3_, while VOC has a lower factor of 0.18. In the terrestrial acidification category, SO_2_ is the reference pollutant (factor = 1), NH_4_ has a factor of 1.96, and NOx has a factor of 0.36. As to the scarcity of fossil resources, crude oil has a factor of 1 kg of oil‐eq, natural gas 0.84 kg of oil‐eq and coal 0.30 kg of oil‐eq.

**TABLE 5:** Classification and characterization of midpoint level indicator.

Emission type derived from LCI	Midpoint level indicator	Unit of the Midpoint level indicator	Characterization factor
CO_2_	Climate change (GWP)	kgCO_2_eq	1
CO			5.53
CH_4_			2.62E+01
NOx			3.67E+02
NOx	Fine particulate matter formation	kgPM10eq	0.47
SO_2_			1.26
PM			4.99
NH_3_			0.26
NO_X_	Photochemical ozone formation	kgNMVOeq	1
VOC			0.18
NOx	Terrestrial acidification	kgSO_2_eq	0.36
SO_2_			1
NH_4_			1.96
Crude oil	Fossil resource scarcity	kgOileq	1
Natural gas			0.84
Coal			0.3

### 5.3 Classification and Characterization at Endpoint Level

Each impact category has a fixed midpoint to endpoint factor, and the endpoint level impact is calculated by multiplying the midpoint level impact amount by a midpoint to endpoint level conversion factor. ReCiPe provides the conversion factor to convert the midpoint level impact into the endpoint level impact. The study's environmental impact at the endpoint indication is shown in Table 6 [66]. The midpoint to endpoint environmental flows are shown in this table, as part of the ReCiPe impact assessment framework. The three cultural viewpoints (Individualist, Hierarchist, Egalitarian) capture the potential effects of environmental pressures on human health, ecosystem quality and resource scarcity through the endpoint indicators. The characterization factors of climate change for human health damage vary between 8.10 × 10‐8 and 1.30 × 10‐5 DALY per kgCO_2_‐eq, with the Egalitarian perspective being the highest because it takes into account the long‐term effects. All perspectives showed a direct effect on human health with a consistent value of 6.30 × 10^−4^ DALY for fine particulate matter formation. Photochemical ozone formation has a similar factor of 9.10 × 10^−7^ DALY lost to human health damage. Ecosystem quality has characterization factors of 1.30 × 10^−7^ species·year for photochemical ozone formation and 2.10×10^−7^ species·year for terrestrial acidification, with a much larger impact of climate change being seen from the Individualist to the Egalitarian perspective, indicating greater concern for long‐term ecological damage. Fossil resource depletion is expressed in monetary units (USD) in the resource scarcity category, where crude oil has the highest characterization factor (0.46 USD per kg oil‐eq), followed by natural gas (0.30 USD per kg oil‐eq) and coal (0.03 USD per kg oil‐eq). These conversion factors allow the midpoint impacts to be aggregated to endpoint impacts and thus provide an overall assessment of the environmental impacts of the diesel engine life cycle.

**TABLE 6:** Conversion factor to convert the midpoint impact into the endpoint impact.

**Endpoint Indicator**	**Midpoint Level Indicator**	**Characterization factor**		
		**Individualist**	**Hierarchist**	**Egalitarian**
Human health (Daily)	Climate change (GWP)	8.10E‐08	9.30E‐07	1.30E‐05
	Fine particulate matter formation	6.30E‐04	6.30E‐04	6.30E‐04
	Photochemical ozone formation	9.10E‐07	9.10E‐07	9.10E‐07
Ecosystem quality (Year)	Photochemical ozone formation	1.30E‐07	1.30E‐07	1.30E‐07
	Terrestrial acidification	2.10E‐07	2.10E‐07	2.10E‐07
	Climate change (GWP)	5.30E‐10	2.80E‐09	2.50E‐08
Resource scarcity (USD)	Fossil resource scarcity by Oil	0.46	0.46	0.46
	Fossil resource scarcity by Coal	0.03	3.00E‐02	3.00E‐02
	Fossil resource scarcity by Natural Gas	3.00E‐01	3.00E‐01	3.00E‐01

Endpoint impacts are aggregated into a single index using the weighing scale, following the work of Martin et al. [118], using ReCiPe for all three perspectives given in Table 7.

**TABLE 7:** Weighing Scale for Endpoint Impacts.

**Perspective**	**Human Health**	**Ecosystems**	**Resources**
Individualist	0.55	0.25	0.2
Hierarchist	0.3	0.4	0.3
Egalitarian	0.3	0.5	0.2

LCIA is the third and major process of the LCA, according to ISO 14042. ISO 14042 provides detailed guidelines for performing the LCIA. There are various methodologies for this step developed by different institutes and research organizations. The main methodologies include CML, IPCC, Recipe, TRACI, and many more. Some of them provide guidelines to assess the impact at the midlevel, while others also provide endpoint indicators. Recipe methodology is suggested and utilized by various researchers as it provides an assessment method for endpoint indicators and is recognized at the global level. Therefore, the impact evaluation approach used is ReCipe. It assesses indicators at two levels: midpoint and endpoint indicators. The midpoint indicators address individual environmental problems such as climate change, eutrophication, and acidification. In contrast, endpoint indicators demonstrate the overall environmental effect on three broader levels: human well‐being, ecosystems, and resource shortages [120].

The environmental emissions, in the form of gases, liquids, and solids, obtained as data in the previous step, i.e., life cycle inventory, are used as input. There are three steps of life cycle assessment, i.e., Classification, Characterization, and Normalization, which were followed closely [25]. Five impact categories were considered for impact assessment: CCP, TAP, FPMFP, POFP, and Fossil resource scarcity. This study used science‐based conversion factors, known as characterization factors, to change and aggregate the LCI outcomes into representative measures of effects on human well‐being, environmental quality, and resource scarcity. Normalization is a technique used to standardize indicator data so that it may be easily compared across different effect categories using a certain reference value [56, 120]. The environmental consequences of various processes were meticulously examined, necessitating the use of a logarithmic scale with Python to manage a wide range of data values. CCP is the mid‐level impact, measured in kgCO_2_eq. GHGs like CO_2_, CH_4_, CO, NO_X,_ etc, are responsible for this impact. Notably, the utilization phase of the engine's life cycle had the highest CCP, which is 359170.95 kgCO_2_eq (98.8%). This is due to the energy‐intensive production of diesel fuel and emissions during engine operation (CO_2_, CH_4_, CO, NOx). The total CCP for the case of pure diesel use is 363513.60 kgCO_2_eq.

The Acidification Potential (AP) also has a substantial effect during the usage phase, estimated at 347.07kgSO_2_eq (97.25%). AP is driven by SO_2_ emissions from diesel fuel production and NO_X_ emissions during engine operation. Due to higher SO_2_ and NOx emissions, aluminium production had a greater AP impact despite its lower mass. Transportation of raw materials and recycling had a lower AP impact. Fossil resource scarcity impact is driven by the consumption of crude oil, coal, and natural gas. In the engine life cycle, it is estimated as 58358.5kgOil‐eq in the case of pure diesel use as fuel. Again, the use phase has the highest value, which is about 95.50% in the case of pure diesel.

**FIGURE 8:**
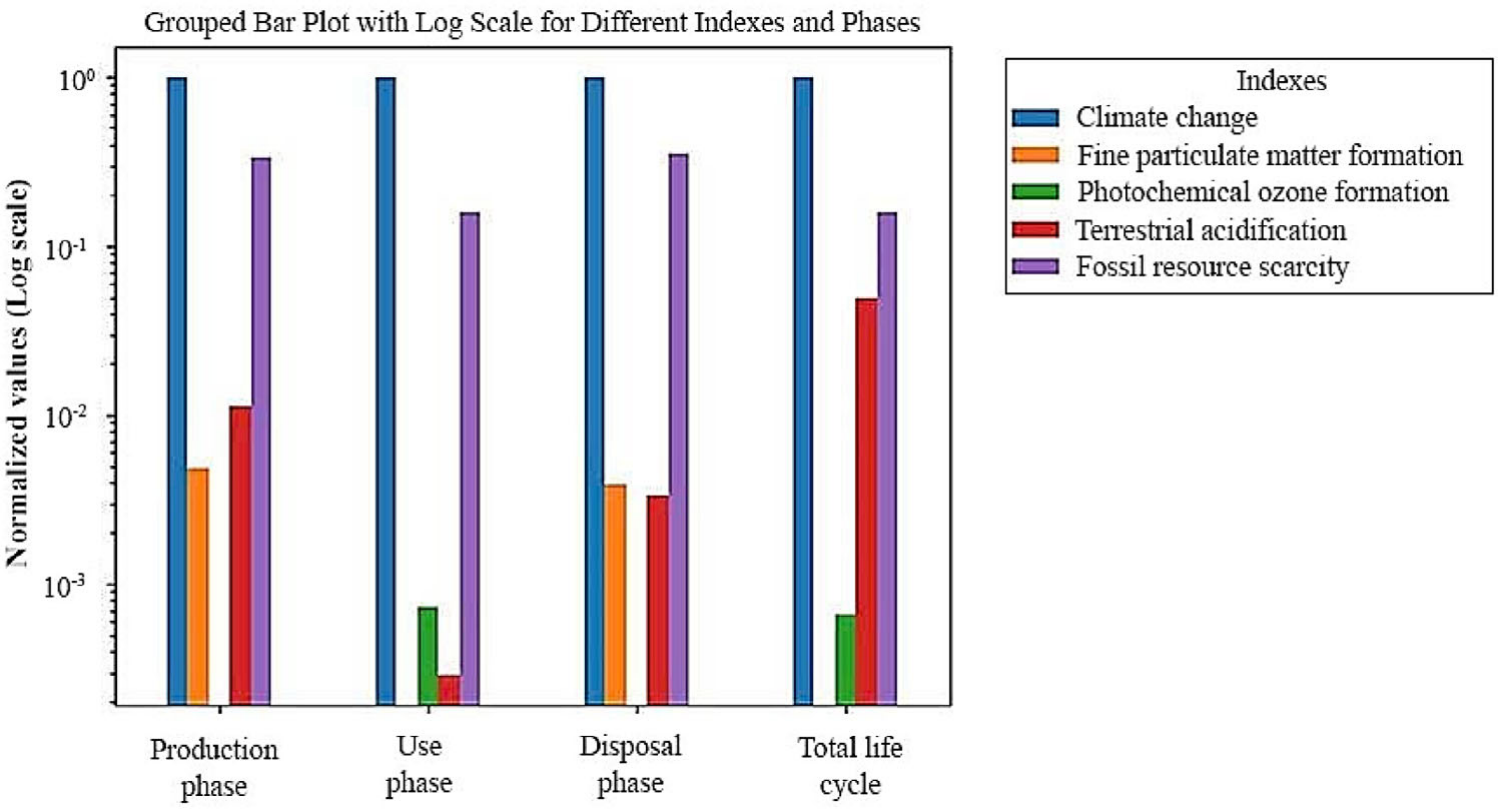
Life Cycle Impact in the case of pure diesel use.

Photochemical ozone formation (POFP) has a significant impact on the engine life cycle. Its amount was estimated as 501.44kgNO_X_‐eq in the case of pure diesel use. Raw materials transportation was second, and alloy production also had a significant POFP impact due to increased CO_2_ emissions. Fine particulate matter formation is caused by particulate matter present in the environment. When PM2.5 particles are breathed, they can reach the upper airways as well as the lungs, leading to adverse health effects in humans. Secondary PM2.5 aerosols are formed in the air from emissions of SO_2_, NH_3_, and NOx, among other elements. It has the greatest impact in the use/operation phase of the engine, followed by the engine production phase. In the use phase, 232.92 kgPMeq of pure diesel combustion is produced. In Table 8, the values of mid‐point life cycle impact are compiled for the case of pure diesel use as fuel. The environmental impact is also depicted graphically in Figure 8.

**TABLE 8:** Mid‐point life cycle impact in the case of pure diesel use.

**Parameters**	**Impact on the Production Phase of the Engine**	**Impact in Use Phase for Pure Diesel**	**Impact on Disposal Phase**	**Total Life Cycle Impact**
Climate change (GWP)	3551.27	359170.95	791.38	363513.60
Fine particulate matter formation	21.87	232.92	4.37	259.16
Photochemical ozone formation	4.67	495.44	1.334	501.44
Terrestrial acidification	44.67	337.55	3.96	18193.93
Fossil resource scarcity	1180.56	56901.04	276.91	58358.51

**FIGURE 9:**
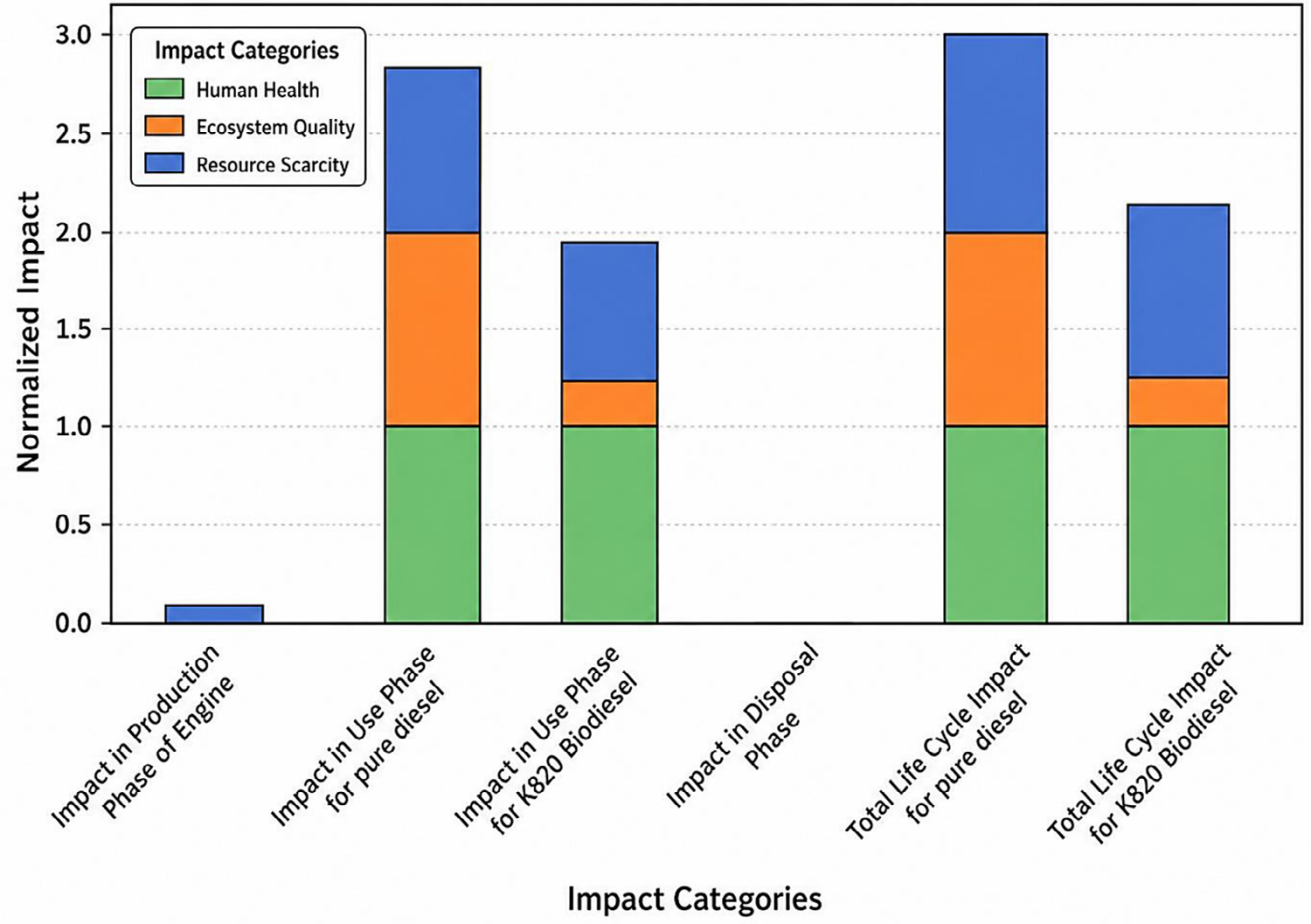
Conversion of endpoint level impacts to a single index.

The midpoint level environmental impacts are converted to the endpoint level impacts according to the Recipe methodology Characterization factor provided in the Recipe. There are three endpoint‐level impacts, which include human health, ecosystem quality (species.yr), and resource scarcity (dollar). Further, the endpoint‐level impacts are converted into a single‐point index using the weights of the impact provided in the recipe [26]. There are three perspectives, namely, Individualist, Hierarchist, and Egalitarian, for consideration of the weights of impacts based on the period. The time horizon for the Egalitarian perspective is explicitly taken as 1,000 years, while for Individualists and Hierarchists, it is 20 and 100 years. In this study, the Egalitarian perspective is used to cover all environmental impacts [118]. The end‐point level life cycle impact, as indicated by a single‐point index of ICE, is depicted in Table 9. In addition, Figure 9 shows the single‐point index of the life cycle impact for all three phases of the ICE.

**TABLE 9:** Endpoint‐level life cycle impact of ICE.

**Parameters**	**Human Health**	**Ecosystem Quality**	**Resource Scarcity**	**Single Index**
Impact on the production phase of the engine	2.1374	0.0029	104554.00	104556.14
Impact on the use phase for pure diesel	144.3658	2.4403	831361.23	831508.01
Impact on the disposal phase	1.0471	0.0016	23347.6125	23348.6612
Total life cycle impact for pure diesel	147.5503	2.4448	959262.16	959412.16
Total life cycle impact for KB20 biodiesel	147.7052	0.5529	876126.72	876275

There are some limitations that exist in every research due to various constraints; however, one research also opens multiple ways of future research in different directions, using contemporary resources and environment. One of the major constraints in life cycle analysis is data availability. At various stages, we have to assume some approximate values. Within the system boundary, considering every operation and material across the phases of material extraction, manufacturing, utilization, and disposal was very difficult, so some operations and materials with little impact were not considered. In an LCA study, there are so many constituents of environmental pollution, each of which is very difficult to measure, so the major constituents of air, water, and solid pollutants are used in this analysis. There are so many environmental impact categories at the midpoint level, and not all of them are easy to handle. Furthermore, various LCA software like SimaPro, GaBi, OpenLCA, GitHub, and GREET are available, and the use of different software can give different outputs. In this work, a diesel engine is taken as a unit to assess its environmental footprint, which is supposed to be the major global problem of environmental pollution in the present times. While there are some other sources of environmental pollution, like gasoline engines. In addition, battery electric vehicles have zero tailpipe emissions; however, the electrical energy is produced with the use of fossil fuels, and the problem of pollution only shifts location. Therefore, the application of LCA can be applied to the power plants. Consideration of different manufacturing methods for engine parts, the use of different materials, and the location diversity of the extraction of materials and manufacturing of parts can give dissimilar results.

## 6 Conclusions

In this study, a Life Cycle Assessment (LCA) for a generic diesel engine manufactured in India was performed successfully. The precise environmental effects of a newly designed diesel engine were evaluated. The research carefully investigated the environmental impact of a newly developed diesel engine, with a focus on CCP, which has a substantial influence on every stage of the diesel engine's life cycle. A life cycle inventory has been performed for various stages, i.e., engine production, operation, and disposal. The Recipe technique was used to examine life cycle impacts, which determines indicators at two tiers: midpoint and endpoint indicators. Midpoint indicators concentrate exclusively on specific issues related to the environment, such as climate change, eutrophication, and acidification. The following are the main outcomes of the study:

- Endpoint indicators indicate the environmental effect on three higher aggregate levels, influencing the well‐being of humans, the ecosystem, and the shortages of resources.
- Five significant environmental impacts are selected for life cycle impact assessment: CCP, PCOF, AP, fine particulate matter formation, and fossil resource scarcity.
- CCP has the highest environmental impact (363513.60 kgCO2eq), followed by fossil resource scarcity (58358.51 kgOileq) and AP (18193.93 kgSO2eq).
- POFP and fine particulate matter formation contribute significant impacts, with 259.16 kgNOX‐eq and 501.4492 kgPMeq in the case of pure diesel usage.
- The engine's utilization phase represents the most energy‐intensive and ecologically destructive part of its life cycle.
- The primary energy demand during the usage phase is closely linked to diesel fuel synthesis, while CCP, AP, FPMF, and POFP are related to engine operation processes.
- Biofuels will play a significant role in meeting future energy demands.

All studies leave open several avenues for further investigation into previously uncharted territory. As we need more sources of biofuel production to meet future energy demands, more researchers can build on the current study by using more biodiesel feedstocks. Additionally, researchers can broaden their scope to include more environmental impacts, such as the potential for toxicity, abiotic depletion, land use, and eutrophication. Additional impact categories, like eutrophication and ozone depletion, can be taken into consideration.

**Nomenclature**
Symbol/Abbreviation: Description
a(i,k,p): Amount of emission *i* from process *k* in phase *p*, kg
AP: Acidification Potential, kg SO_2_‐eq
BEV: Battery Electric Vehicle, —
BOD: Biological Oxygen Demand, kg
CCP (or GWP): Climate Change Potential (Global Warming Potential), kg CO_2_‐eq
CF(i,c): Characterization factor of emission *i* for impact category *c*, Varies
CF(c→d,v)EP: Midpoint‐to‐endpoint conversion factor, Varies
CH_4_
: Methane, kg
CO: Carbon Monoxide, kg
CO_2_
: Carbon Dioxide, kg
COD: Chemical Oxygen Demand, kg
CRDi: Common Rail Direct Injection, —
DALY: Disability Adjusted Life Year, DALY
dyear: Number of operating days per year, day yr^−1^
D(d,v): Endpoint damage indicator, Varies
E(i,p): Emission of pollutant *i* in phase *p*, kg
E(i, LC): Total life‐cycle emission of pollutant *i*, kg
EGR: Exhaust Gas Recirculation, —
EV: Electric Vehicle, —
FPMF: Fine Particulate Matter Formation, kg PM_10_‐eq
GDI: Gasoline Direct Injection, —
GHG: Greenhouse Gas, —
GWP: Global Warming Potential, kg CO_2_‐eq
HCCI: Homogeneous Charge Compression Ignition, —
HCl: Hydrogen Chloride, kg
H_2_S: Hydrogen Sulfide, kg
Icmid(p): Midpoint impact of category *c* in phase *p*, Varies
Icmid(LC): Total life‐cycle midpoint impact, Varies
Icnorm: Normalized midpoint impact, —
Icref: Reference value for normalization, Varies
ICE: Internal Combustion Engine, —
ICEV: Internal Combustion Engine Vehicle, —
IPCC: Intergovernmental Panel on Climate Change, —
ISO: International Organization for Standardization, —
k: Process index, —
LCA: Life Cycle Assessment, —
LCI: Life Cycle Inventory, —
LCIA: Life Cycle Impact Assessment, —
MPFi: Multi‐Point Fuel Injection, —
NH_3_
: Ammonia, kg
NH_4_^+^
: Ammonium, kg
NMVOC: Non‐Methane Volatile Organic Compounds, kg NMVOC‐eq
NOx: Nitrogen Oxides, kg
p: Life‐cycle phase index, —
PCCI: Premixed Charge Compression Ignition, —
PM: Particulate Matter, kg
PM_10_
: Particulate Matter with aerodynamic diameter <10 µm, kg
POFP: Photochemical Ozone Formation Potential, kg NMVOC‐eq
RCCI: Reactivity Controlled Compression Ignition, —
ReCiPe: Life Cycle Impact Assessment Method, —
r(j,k,p): Quantity of resource *j* consumed in process *k* and phase *p*, kg
R(j, LC): Total life‐cycle resource consumption, kg
Sv: Aggregated endpoint score, Points
SO_2_
: Sulfur Dioxide, kg
VOC: Volatile Organic Compounds, kg
VVT: Variable Valve Timing, —
w(d,v): Weighting factor for damage category *d*, —
Y: Engine lifetime, year

## Conflicts of Interest

The authors declare no conflicts of interest.

## Data Availability

Data will be made available upon request.
